# Typhoid Fever in Missouri

**Published:** 1845-07

**Authors:** 


### TYPHOID FEVER IN MISSOURI.

In a letter to the editors, dated June 2d, Dr. Z. T. Robards, of Grand Pass, Missouri, mentions the prevalence of a fatal fever in his neighborhood during the past winter. Some of the facts communicated will be found interesting. He says:—

"It may not be improper to remark that a typhoid fever prevailed in the vicinity of Jefferson City during the last winter, which has proved unusually fatal. The characteristic symptoms are those of typhus gravior; the patient appears much in the condition of one intoxicated from ardent spirits even from the beginning. The patients complained of acute pain at the junction of the occiput and atlas, which was much increased upon the slightest rotation of the head from side to side. In a *post-mortem* examination which I witnessed, there was matter found at the base of the brain, and commencement of the spinal column, beneath the tunica arachnoides. There were likewise symptoms of inflammation in the serous tissues of the abdomen generally. In this disease the pulse fails upon withdrawing the smallest amount of blood. My only object in mentioning this fact is to ascertain if any analogy exists between this and the epidemic reported to be prevailing in your vicinity. Some of the cases were attended with severe diurnal rigors, or chills, and terminated fatally on the third day of the attack. I have been informed that they presented the same appearances on *post-mortem* examination which have been mentioned."

Dr. R. alludes to Erysipelas, of the prevalence of which in this city he speaks of having heard. No epidemic erysipelas has prevailed in Louisville. Cases have appeared occasionally in private practice, and at one time in the spring surgical operations were pretty sure of being followed by erysipelatous inflammation, but the disease has never been general in this place. For nearly two years a strong predisposition to it has existed among the patients of the Marine Hospital, and so great at present does it appear to be, that it has been deemed advisable by the Hospital Committee to direct that the subjects of this complaint be taken to the Pest house. The health of the city is good. Y.

